# Inhibitory properties of Chinese Herbal Formula SanHuang decoction on biofilm formation by antibiotic-resistant Staphylococcal strains

**DOI:** 10.1038/s41598-021-86647-8

**Published:** 2021-03-30

**Authors:** Shaoe Zhang, Peizhao Wang, Xiaotao Shi, Honglue Tan

**Affiliations:** Henan Orthopedic Institute, Henan Luoyang Orthopedic-Traumatological Hospital (Henan Orthopedic Hospital), Qiming Southern Road, Luoyang, 471002 Henan People’s Republic of China

**Keywords:** Microbiology, Bacteria, Biofilms

## Abstract

The aim of this study was to explore the effect of Chinese herbal SanHuang decoction (SH) on biofilm formation of antibiotic-resistant *Staphylococci* on titanium surface, and to explore its mechanism. Biofilm-forming ATCC 35984, ATCC 43300 and MRSE 287 were used in this study. The MICs of SH and vancomycin against *Staphylococci* were determined by the broth microdilution method. Six groups were designed, namely control group (bacteria cultured with medium), 1/8MIC SH group (1MIC SH was diluted by 1/8 using TSB or saline), 1/4MIC SH group, 1/2MIC SH group, 1MIC SH group and vancomycin group (bacteria cultured with 1MIC vancomycin). The inhibitory effect on bacterial adhesion and biofilm formation were observed by the spread plate method, CV staining, SEM, and CLSM. Real-time PCR was performed to determine the effect of SH on the expression levels of *ica* AD and *ica* R gene in ATCC 35984 during the biofilm formation. The strains were found to be susceptible to SH and vancomycin with MIC of 38.75 mg/ml and 2.5 μg/ml, respectively. SH with 1 MIC and 1/2 MIC could inhibit the bacteria adhesion, showing only scattered adhesion from SEM. CLSM showed that SH with 1 MIC and 1/2 MIC inhibited the biofilm formation. The quantitative results of the spread plate method and CV staining showed that there was significant differences between the SH groups (*P* < 0.05). Further, with an increase in SH concentration, the inhibitory effect became more obvious when compared with control group. Among the groups, vancomycin had the strongest inhibitory effect on bacterial adhesion and biofilm formation (*P* < 0.01). With an increase in SH concentration, the expression levels of *ica* AD decreased, and the expression of *ica* R increased correspondingly (*P* < 0.05). In conclusions, SH can inhibit the biofilm formation of antibiotic-resistant *Staphylococci*. Its probable mechanistic activity may be through the inhibition of polysaccharide intercellular adhesin synthesis by down-regulating the expression of *ica* AD gene.

## Introduction

Postoperative infection is one of the serious complications impacting the clinical results of orthopedic surgery, and its inadequate management will lead to nonunion of fracture, loss of limb function and even amputation, which will inevitably impose an economic burden to the patients’ families and social medical insurance^1^. According to the literature, the incidence of postoperative infections for closed fracture is about 1–2%, but the incidence of an open fracture is significantly as high as 30%^1,2^. Studies have shown that 80% of clinical infections are related to bacterial biofilm formation, which plays an important role in the pathogenesis of orthopedic infections^3^. Firstly, bacteria in a biofilm are very resistant to antibiotics, and their antibiotic tolerance is 1000-fold higher than the MIC (minimum inhibitory concentration) of the planktonic form^3,4^. Secondly, the biofilm and its colonized pathogens can successfully defend the host’s immune system, so that they cannot be phagocytized and cleared by polymorphonuclear leukocytes^5^.

Titanium and its alloys have superb biocompatibility, low elastic modulus, and favorable corrosion resistance. These exceptional properties lead to its wide use as a medical implant material for orthopedic surgery^6^. Titanium itself does not have antibacterial properties, so bacteria can gather and adhere to its surface resulting in biofilm^6^. Hence, how to inhibit biofilm formation on the surface of titanium alloy becomes important in the prevention and treatment of orthopedic infections.

For intractable orthopedic infections, in addition to local debridement, an important option is a long-term application of systemic intravenous antibiotics^7^. In clinical practice, a very large proportion of implant-related infections are caused by *Staphylococci*, and two *Staphylococcal* species, *Staphylococcus aureus* and *Staphylococcus epidermidis*, together account for two out of three infection isolates^8^. As for biofilm-related infections, however, antibiotic-resistant *Staphylococcus*, especially MRSE (meticillin-resistant *Staphylococcus epidermidis*) and MRSA (methicillin-resistant *Staphylococcus aureus*), makes the treatment of implant-associated infections with antibiotics still one of the challenges for orthopedic surgeons^2,4,7^. Hence, discovering alternative treatment methods for orthopedic infections linked to multi-antibiotic-resistant bacterial biofilm is becoming more and more relevant.

For a long time, traditional Chinese medicine (TCM) has been widely used in the treatment of various infectious diseases in China, and many traditional or empirical prescriptions have been proved to have satisfactory clinical therapeutic effects^9–12^. The SanHuang decoction (abbreviated as SH) is one of the treatment methods widely used in our hospital for treatment of limb infections with bone and implant exposure. This decoction is composed of *Scutellaria baicalensis Georgi*, *Coptidis rhizoma*, *Cortex Phellodendri chinsis* and other Chinese herbs. According to the theory of traditional Chinese medicine, it has the heat-clearing and detoxing functions. Through external soaking and washing of the locally infected wound with SH, it was found that this decoction can effectively control the infections^10–12^. However, there has been no study to date that has explored the antibiofilm formation properties of the SH in vitro.

Therefore, based on previously published clinical studies and the widely used titanium implant material for orthopedic surgery^6,10–12^, the aim of this study was to investigate the effect of SH decoction on adhesion and biofilm formation of MRSE and MRSA on titanium surfaces, and also to explore the probable mechanism behind its effect on biofilm formation.

## Materials and methods

### Preparation of SH decoction

SH decoction contains *Scutellaria baicalensis Georgi* (25 g), *Coptidis rhizoma* (25 g), *Cortex Phellodendri chinsis* (25 g), *Sophora flavescens* (20 g), *Lonicera Japonica* (20 g), *Forsythia suspensa* (20 g) and *Taraxacum mongolicum Hand.-Mazz.* (20 g) (Table 1). All the herbs were provided by the TCM pharmacy of Henan Luoyang Orthopedic-Traumatological Hospital following the requirement standards of the section one of Pharmacopoeia of the People's Republic of China (2015 edition). The procedure of preparing the SH aqueous extract is as follows: firstly, all herbs were tipped into a ceramic pot with 1000 ml pure water and soaked for 30 min. Secondly, the concoction was boiled at high heat for 30 min, and was maintained at a low flame until the aqueous extract reduced to 250 ml. Finally, aqueous extract was filtered with filter paper, and water extraction of the liquid was performed through Ø 22 μm filter. Based on the original herb dose, the initial liquid concentration of the extract solution was about 0.62 g/ml, which was stored in the refrigerator at − 20 °C for the subsequent experiments. Only one batch of SH were used in this study.

**Table 1:** The composition of SH decoction.

Chinese name	Latin name	Part used	Proportion (g)
Huang Qin	*Scutellaria baicalensis Georgi*	Root	25
Huang Lian	*Coptidis rhizoma*	Root	25
Huang Bai	*Cortex Phellodendri chinsis*	Bark	25
Ku Shen	*Sophora flavescens*	Rhizome	20
Jin Yin Hua	*Lonicera Japonica*	Bud	20
Lian Qiao	*Forsythia suspensa*	Fruit	20
Pu Gong Ying	*Taraxacum mongolicum Hand.-Mazz*	Root	20

### Preparation of bacterial strains

Two standard strains, the positive biofilm-forming *S. epidermidis* ATCC 35984 (American Type Culture Collection [ATCC], MRSE) and *S. aureus* ATCC 43300 (MRSA), and one clinical isolate, methicillin-resistant *S. epidermidi*s (MRSE 287) which was isolated from one patient with an orthopaedic implant-related infection, were used in this study^13,14^. The strains frozen in a − 80 °C refrigerator were rapidly thawed and inoculated on a Tryptone Soy Agar (TSA) plate and cultured in an incubator at 37 °C for 24 h. A single colony was selected from the TSA and inoculated in a 50 ml centrifuge tube containing 10 ml Tryptone Soy Broth (TSB), the tube was incubated for 10 h at 37 °C with agitation at 100 rpm. Then, 25 µl of bacterial suspension was transferred to another sterile centrifuge tube containing 10 ml TSB and incubated at 37 °C for 14 h, after which 1 ml suspension was transferred into a 1.5 ml centrifuge tube and the bacteria in the suspension was harvested by centrifugation for 5 min at 4 °C and 1000 rpm (Sorvall TC6 centrifuge, Du Pont, Bad Nauheim, Germany). The precipitate was then washed three times with 0.15 M PBS (phosphate buffered saline) to remove the remaining TSB, and resuspended in sterile PBS to an optical density of 0.490 at 600 nm using Synergy HT multi-detection microplate spectrophotometer (Bio-tek, Winooski, VT), which corresponded to 10^9^ CFUs (Colony forming units)/ml.

### Minimum inhibitory concentration (MIC) determination

The broth microdilution method was used to determine the MIC of SH, gentamicin, penicillin and vancomycin as recommended by the National Committee for Clinical Laboratory Standards Institute^15^. An overnight culture of the strains were diluted tenfold in TSB and incubated at 37 °C until they reached exponential growth phase. Serial twofold dilutions of SH (155 mg/ml to 4.84 mg/ml), gentamicin (1024 μg/ml to 0.5 μg/ml), penicillin (1024 μg/ml to 0.5 μg/ml) and vancomycin (8 μg/ml to 0.0625 μg/ml) in Mueller Hinton (MH) broth were prepared in a 96-wells plate with 190 μl per well. Ten microlitres of bacterial inocula of each strain containing 5 × 10^6^ CFUs/ml was added in all wells. A number of wells with only MH broth (without inoculum and drugs) in each plate were designed as blank control, and a number of wells with MH broth and inoculum (without drugs) were designed as negative control. The plates were incubated for 24 h at 37 °C. The MIC was detected following addition of 50 μl of INT (2-4-Iodophenyl-3-4-nitrophenyl-5-phenyl-2H-tetrazolium chloride) at a final concentration 0.2 mg/ml in all the wells and incubated for 30 min at 37 °C. Bacterial growth was determined by observing the color change of INT in the wells. Biologically active bacterial cells will reduce the colourless tetrazolium salt which act as an electron acceptor to a red-coloured formazan product^16^. Inhibition of bacterial growth is observed when the solution in the well remained clear after incubation with INT. MIC was defined as the lowest extract concentration that completely inhibits the growth of microorganisms. Each experiment was performed three times in duplicate.

### Experimental grouping

In total, our study had six experimental groups, namely control group (bacteria cultured with only TSB or saline), 1/8MIC SH group (1MIC SH was diluted by 1/8 using TSB or saline, SH concentration was 4.85 mg/ml), 1/4MIC SH group (SH concentration was 9.69 mg/ml), 1/2MIC SH group (SH concentration was 19.38 mg/ml), 1MIC SH group (SH concentration was 38.75 mg/ml) and vancomycin group (bacteria cultured with TSB or saline containing vancomycin with 1MIC, vancomycin concentration was 2.5 µg/ml).

### Effect of different SH concentration on bacterial adhesion and biofilm formation

Titanium discs of 10 mm in diameter and 1 mm in thickness were placed into a 48-well plate (Costar 3548, Corning, NY, USA), and six replicates were used for each group. All disks were autoclaved at 121 °C for 20 min before used. *S. epidermidis* ATCC 35984, *S. aureus* ATCC 43300 and MRSE 287 were diluted to a density of 10^6^ CFUs/ml with 0.9% saline, saline-vancomycin, or different sub-MICs of SH (dilution with saline), and 1 ml of the cell suspension was added to the well containing the discs. The plates were incubated in aerobic conditions for 4 h. Then, the bacterial adhesion on the titanium surface were analyzed with the following methods.

#### The spread plate method

The discs were gently washed three times with saline to remove loosely adherent bacteria, and then transferred into a 10 ml glass tube containing 0.5 ml saline. The tubes were then placed in an ultrasonic bath (B3500S-MT, Branson Ultrasonics Co., Shanghai, China) and the bacteria attached on the disc were dislodged by ultrasonication (5 min) at a frequency of 50 Hz. Ultrasonication was followed by rapid vortex mixing (Vortex Genie 2, Scientifific Industries, Bohemia, NY, USA) at maximum power for 1 min to remove bacteria that had adhered to the material. This method is known to be effective for removing biomaterial-adherent bacteria^17^. The vortexed solutions were serially diluted tenfold and the final three dilutions were plated in triplicate onto TSA and then incubated at 37 °C for 24 h. Finally, the number of CFUs on the TSA were counted, and the number of bacteria adhering to titanium surface was calculated and is expressed relative to the surface area (CFUs/mm^2^).

#### SEM assay

A sterile pipette was used to carefully remove the saline from each well. The discs were transferred with forceps into another fresh 48-well plate and gently washed with saline three times to remove loosely adherent bacteria. The discs were fixed in 2.5% glutaraldehyde for 2 h at 4 °C, then washed with PBS and dehydrated through a series of graded ethanol solutions (25, 50, 75, 95 and 100%). The samples were subsequently freeze-dried, sputter coated with gold, and observed using SEM (Joel JSM-6310LV, JEOL Ltd., Tokyo, Japan).

After incubation for 24 h with TSB, TSB-vancomycin, or different sub-MICs of SH (dilution with TSB). Biofilm formation on the titanium surface were analyzed by the following methods. Crystal violet (CV) staining assay: A sterile pipette was used to carefully remove the TSB from each well. The discs were transferred into another 48-well plate and gently washed three times with PBS. Then, 1 ml of 2.5% glutaraldehyde solution was added into each well for 10 min to allow fixation. The glutaraldehyde solution was removed and the wells were washed with PBS. Subsequently, PBS was removed and 2 ml of 0.1% (w/v) aqueous CV solution was added to each well followed by incubation for 20 min at room temperature. CV solution was discarded, and the wells were washed with PBS again and air-dried for 12 h in the dark. Next, the quantity of biofilm was analyzed by adding 30% acetic acid in a volume of 1 ml to each well to dissolve dye for 30 min, after which 200 μl of the dye solution were transferred into a 96-well microtiter plate, one group equaling one vertical row of the plate. The optical density was read at a wavelength of 492 nm using a plate reader (Synergy HT, BioTEK)^18^.

#### CLSM assay

After 24 h incubation, the TSB medium were removed carefully from each well, and the discs were transferred into another 48-well plate and gently washed with PBS three times. Then the wells containing the titanium discs were added with 500 μl combination dye (LIVE/DEAD Baclight bacteria viability kits, L13152; Molecular Probes, Eugene, OR, USA) in a dark environment at room temperature for 15 min and were subsequently analyzed with a CLSM (Leica TCS SP2; Leica Microsystems, Heidelberg, Germany). The live/dead kit containing two kinds of fluorescence dyes were used to distinguish the viable and non-viable cells under the fluorescence microscope, because dye SYTO 9 can make the viable bacteria with intact cell membranes display green fluorescence, whereas dye PI (Propidium Iodide) can make non-viable bacteria with damaged membranes display red fluorescence. The Leica confocal software was used to analyze the biofilm images.

### Effect of different incubation times on biofilm formation

The bacterial biofilm formation on the titanium surface was also observed at different incubation time-points. Titanium discs (10 mm in diameter and 1 mm in thickness) were placed into 48-well plates, 1 ml of the cell suspension with 10^6^ CFUs/ml adjusted by TSB, SH at different sub-MICs and TSB-vancomycin were added to each well and incubated at 37 °C for 6, 12, 18, 24, and 48 h. At the specified time-point, the quantitative analysis of bacterial biofilm formation on the titanium surface were observed by CV staining assay, as described above.

### Real-time PCR analysis of *ica* AD and *ica* R transcription

The experiment had six groups, including the TSB, 1/8MIC SH, 1/4MIC SH, 1/2MIC SH, 1MIC SH and vancomycin group. *S. epidermidis* ATCC 35984 was selected for the *ica* AD and *ica* R expression assays. Titanium discs (14 mm in diameter and 1 mm in thickness) were placed into 24-well plates, and 2 ml of the cell suspension with 10^6^ CFUs/ml adjusted by TSB only, SH at different sub-MIC and TSB-vancomycin were added to each well and incubated at 37 °C for 24 h. Then, the bacterial biofilms on the disc surface were harvested together with the planktonic bacteria by sonication and transferred into the RNAprotect bacterial reagent (Qiagen, Germantown, MD, USA) to ensure RNA integrity. Bacteria were pelleted by centrifugation at 4 °C and 8000*g* for 10 min. The protective reagent was removed and resuspended the pellets in 0.8 ml TE buffer (10 mM TrisHCl, 1 mM EDTA, pH 7.0) containing 100 g/ml lysostaphin (Sigma, St Louis, MO, USA). Total RNA was isolated using Axyprep Multisource Total RNA Miniprep Kit (Axygen bioscience, Union City, NJ, USA) according to the manufacturer’s instructions. RNA was reversely transcribed to cDNA using the PrimeScript RT reagent kit (TaKaRa, Shiga, Japan). Real-time PCR was performed on an ABI 7500 Fast machine according to the SYBR Premix Ex Taptm kit (TaKaRa). The reactions were performed using cDNA templates, and with specific forward and reverse primers. The amplification conditions were as follows: 50 °C for 20 s; 40 cycles of 95 °C for 15 s, and 60 °C for 1 min; then 95 °C for 10 min. The expression levels of *ica* AD and *ica* R were evaluated and normalized to the internal standard gene 16S rRNA. The quantification of gene expression was based on the CT value of each sample, which was calculated as the average of three replicate measurements for each sample analyzed. The primers used for the RT-PCR are shown in Table 2^14^.

**Table 2:** Primers used in this study.

Gene	Gene description	Primer sequence (5^′^–3^′^)	
*ica* A	Intercellar adhesion A	Forward	AACAAGTTGAAGGCATCTCC
		Reverse	GATGCTTGTTTGATTCCCT
*ica* D	Intercellar adhesion D	Forward	ATCGTTGTGATGATTGTTTA
		Reverse	GATATGTCACGACCTTTCTT
*ica* R	Intercellar adhesion R	Forward	CCATTGACGGACTTTACCAG
		Reverse	CAAAGCGATGTGCGTAGGA
16S rRNA	Normalizing internal standard	Forward	TCGTGTCGTGAGATGTTGGGTTA
		Reverse	GGTTTCGCTGCCCTTTGTATTGT

### Statistical analysis

All the experiments were performed in triplicate and repeated three times. Data are expressed as mean ± standard deviation. The total number of bacteria was normalized by the number obtained from the control group. This method for presenting the data was chosen because the number of colony forming units was not exactly the same for each condition. The results were tested using a one-way analysis of variance. The Tukey’s multiple comparison test was applied for multiple comparisons between individual treatments once significant difference was observed by ANOVA. The differences observed between groups were considered to be significant at *P* < 0.05 using SPSS 19.0 software.

## Results

### MIC of SH decoction and antibiotics

*Staphylococcus epidermidis* ATCC 35984 (methicillin-resistant *S. epidermidi*s, MRSE), *S. aureus* ATCC 43300 (methicillin-resistant *S. aureus,* MRSE) and MRSE 287 were found to be susceptible to SH decoction with MIC values of 38.75 mg/ml. *S. epidermidis* ATCC 35984, *S. aureus* ATCC 43300 and MRSE 287 exhibited a low level of susceptibility to gentamicin (MIC = 16 μg/ml, 64 μg/ml and 256 μg/ml, respectively) and penicillin (MIC = 128 μg/ml, 32 μg/ml and 128 μg/ml, respectively). Vancomycin was found to possess high inhibitory activity against three bacteria under investigation, with MIC values of 2.5 μg/ml.

### Effect of SH decoction on bacterial adhesion

**Figure 1:**
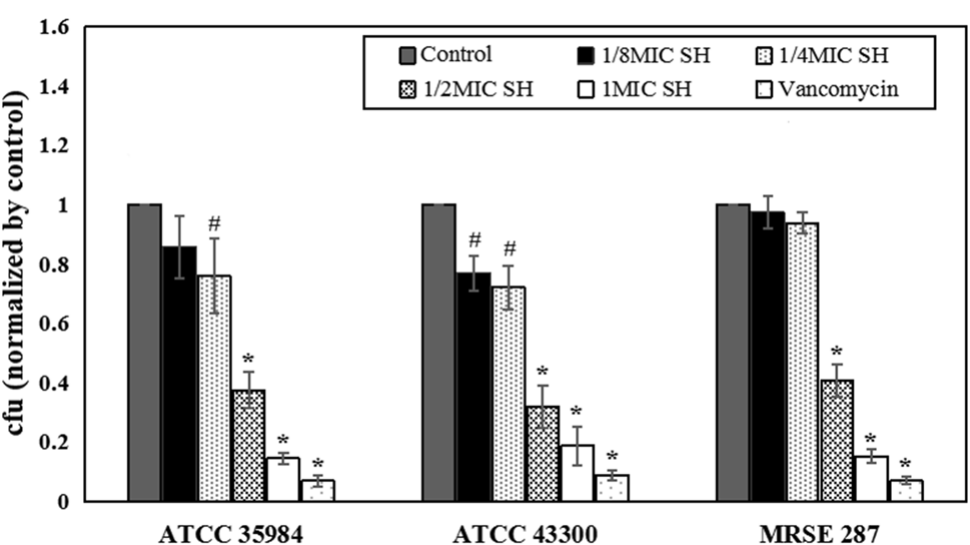
Attachment assay of bacterial strains on titanium surface. Compared with control, 1/8MIC SH and 1/4MIC SH group, *P < 0.01. Compared with control group, ^#^*P* < 0.05.

From the results of the spread plate method (Fig. 1), 1/2MIC SH, 1MIC SH and vancomycin could significantly inhibit the bacterial adhesion on the titanium surface, compared with the control, 1/8MIC SH and 1/4MIC SH (*P* < 0.01). While between the control and 1/8MIC SH group of *S. epidermidis* ATCC 35984, and among the control, 1/8MIC SH and 1/4MIC SH group of MRSE 287, no significant differences were observed (*P* > 0.05). For *S. epidermidis* ATCC 35984, 1/4MIC SH significantly inhibited bacterial adhesion compared with that of the control group (*P* < 0.05). For *S. aureus* ATCC 43300, compared with control group, both 1/4MIC SH and 1/8MIC SH also inhibit the bacterial adhesion (*P* < 0.05); while no significant differences between 1/4MIC SH and 1/8MIC SH groups were found (*P* > 0.05). We also observed statistically significant difference of bacterial adhesion between SH groups under 1/4, 1/2 and 1MIC concentrations, which was more obvious with the increase of SH concentration (*P* < 0.01). On the other hand, vancomycin at 1MIC had the strongest inhibitory effect on the bacterial adhesion among the groups (*P* < 0.01).

**Figure 2:**
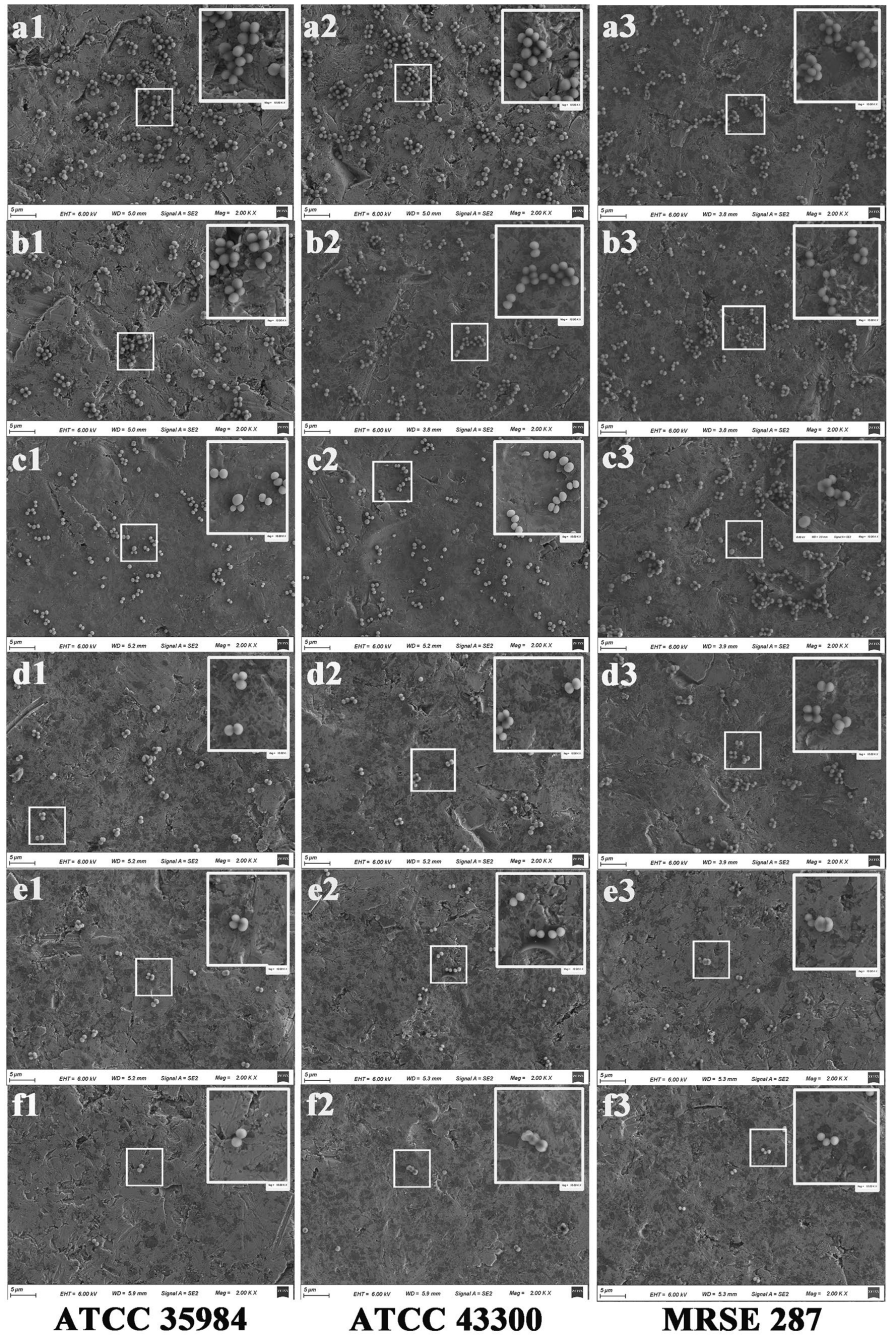
SEM of bacterial adhesion on the titanium surface. Compared with control, 1/8MIC SH and 1/4MIC SH groups, 1/2MIC, 1MIC SH and Vancomycin can obviously inhibit bacterial adhesion. (**a**) Control group. (**b**) 1/8MIC SH group (**c**) 1/4MIC SH group. (**d**) 1/2MIC SH group. (**e**) 1MIC SH group. (**f**) Vancomycin group. The insert photograph is a 5× magnification of the white box in the image. Magnification, ×2000. Scale bars, 5 μm.

Further, we performed SEM (Fig. 2) after 4 h incubation with bacteria and found that scattered bacterial colonies were observed on the titanium surface in the 1/2MIC SH (Fig. d1, d2, and d3), 1MIC SH (Fig. c1, c2, and c3) and vancomycin group (Fig. f1, f2, and f3). However, the bacteria in the control (Fig. a1, a2, and a3), 1/8MIC SH (Fig. b1, b2, and b3) and 1/4MIC SH group (Fig. c1, c2, and c3) obviously clumped together. For SH groups with 1/4, 1/2 and 1MIC concentrations, the inhibition of the bacterial adhesion on the surface of titanium disc became more obvious with the increase of SH concentration. Meanwhile, the number of adherent bacterial colony on the titanium surface in vancomycin group (Fig. f1, f2, and f3) was the least in all groups.

**Figure 3:**
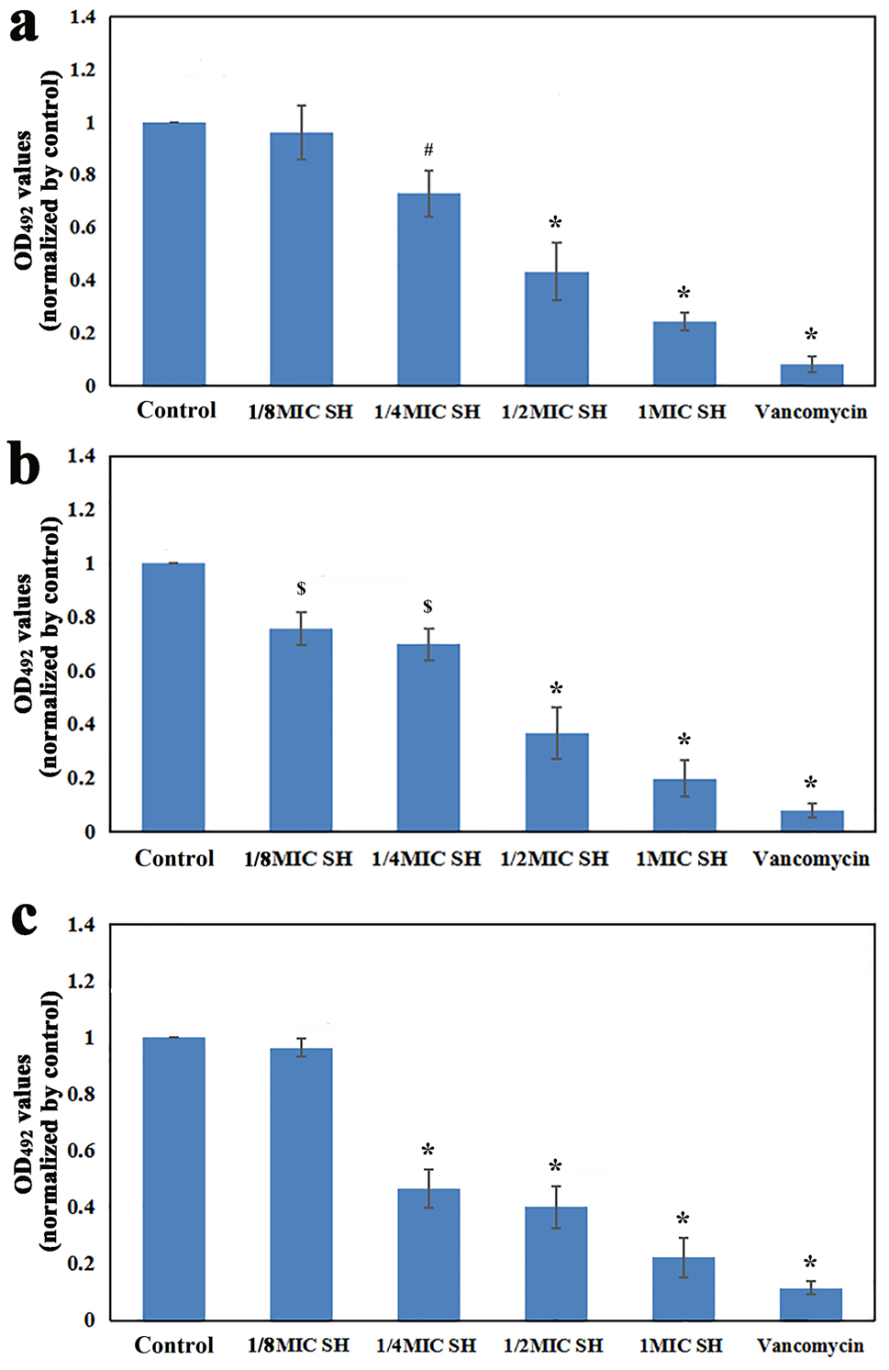
CV staining of bacterial biofilms under different culture conditions. Compared with the control and 1/8MIC SH groups, *P < 0.01. Compared with the control group, ^#^*P* < 0.05. (**a**) *S. epidermidis* ATCC 35984. (**b**) *S. aureus* ATCC 43300. (**c**) MRSE 287.

### Antibiofilm activity of SH decoction

As shown in Fig. 3, compared with the control and 1/8MIC SH groups of *S. epidermidis* ATCC 35984 (Fig. 3a) and MRSE 287 (Fig. 3c), the OD values of 1/4MIC SH, 1/2MIC SH, 1MIC SH and vancomycin groups were significantly decreased (*P* < 0.01), while there was no significant difference between the control and 1/8MIC SH groups (*P* > 0.05). For the SH groups in the two strains, the OD values decreased with the increase of SH concentration, and the difference among the 1/8MIC, 1/4MIC, 1/2MIC and 1MIC SH groups was statistically significant (*P* < 0.05). For *S. aureus* ATCC 43300 (Fig. 3b), compared with the control group, the other groups had lower OD values with significant difference among 1/4MIC SH, 1/2MIC SH, 1MIC SH and vancomycin groups (*P* < 0.01), and on difference between 1/8MIC SH and 1/4MIC SH groups (*P* > 0.05). The OD value of vancomycin group was lower than that of 1MIC SH group (*P* < 0.01).

**Figure 4:**
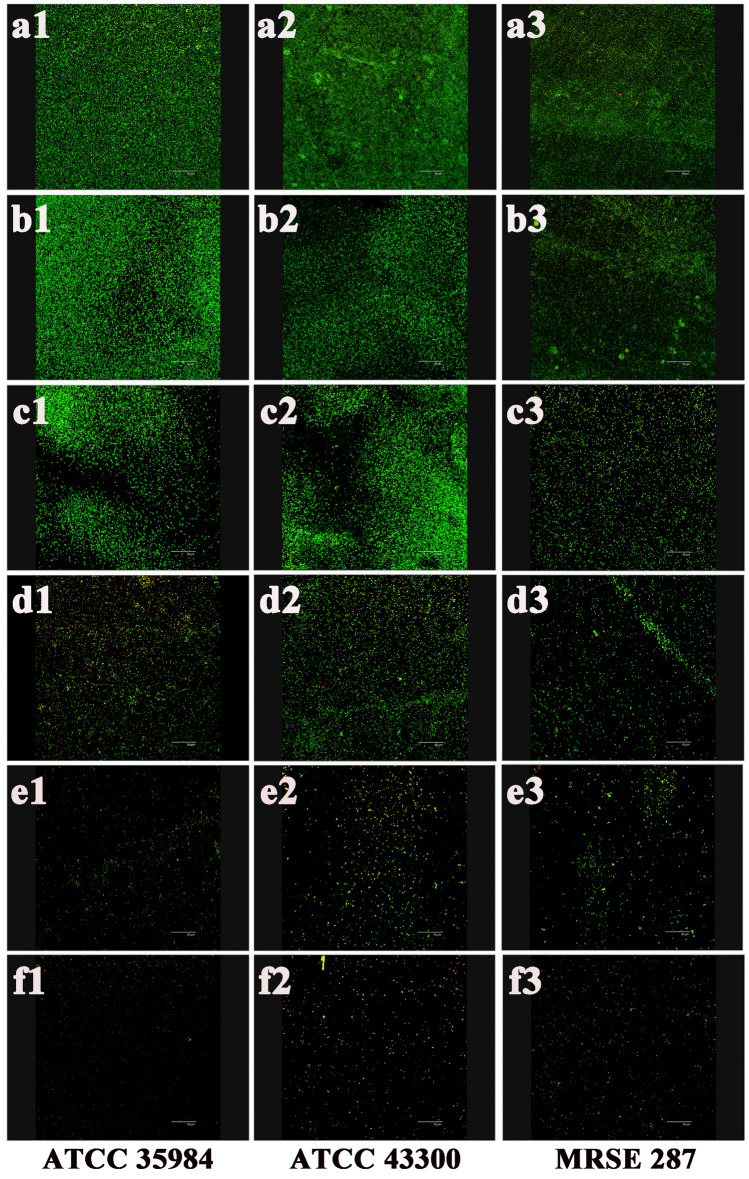
CLSM of biofilm formation on the titanium surface. High-intensity fluorescence was seen in the TSB, 1/8MIC SH, and 1/4 MIC SH groups, and low-intensity fluorescence was seen in the 1/2MIC SH, 1MIC SH, and vancomycin groups. (**a**) Control group. (**b**) 1/8MIC SH group. (**c**) 1/4MIC SH group. (**d**) 1/2MIC SH group. (**e**) 1MIC SH group. (**f**) Vancomycin group. Magnification, ×400. Scale bars, 50 µm.

CLSM of bacterial biofilms on the titanium surface is shown in Fig. 4. After culture for 24 h, a large number of bacterial colonies were clustered to form a dense biofilm structure on the titanium surface in the control (Fig. a1, a2, and a3) and 1/8MIC SH (Fig. b1, b2, and b3) groups, displaying high-intensity green fluorescence. However, with the increase of the SH concentration, the intensity of green fluorescence gradually decreased, suggesting that the bacterial biofilm formation was also gradually inhibited. For *S. epidermidis* ATCC 35984 and *S. aureus* ATCC 43300, high-intensity green fluorescence was still visible in 1/4MIC SH group (Fig. c1 and c2), indicating the existence of severe biofilm formation; While in 1/4MIC SH group of MRSE 287 (Fig. c3) and in 1/2MIC SH groups of three bacterial strains (Fig. d1, d2, and d3), low-intensity green fluorescence was shown, indicating that there was still mild biofilm formation on the titanium surface. In the 1MIC SH (Fig. e1, e2, and e3) and vancomycin (Fig. f1, f2, and f3) groups, only scattered green fluorescence of the bacterial colonies were seen, showing no biofilm formation.

### Effect of SH decoction on biofilm formation at different incubation time-point

**Figure 5:**
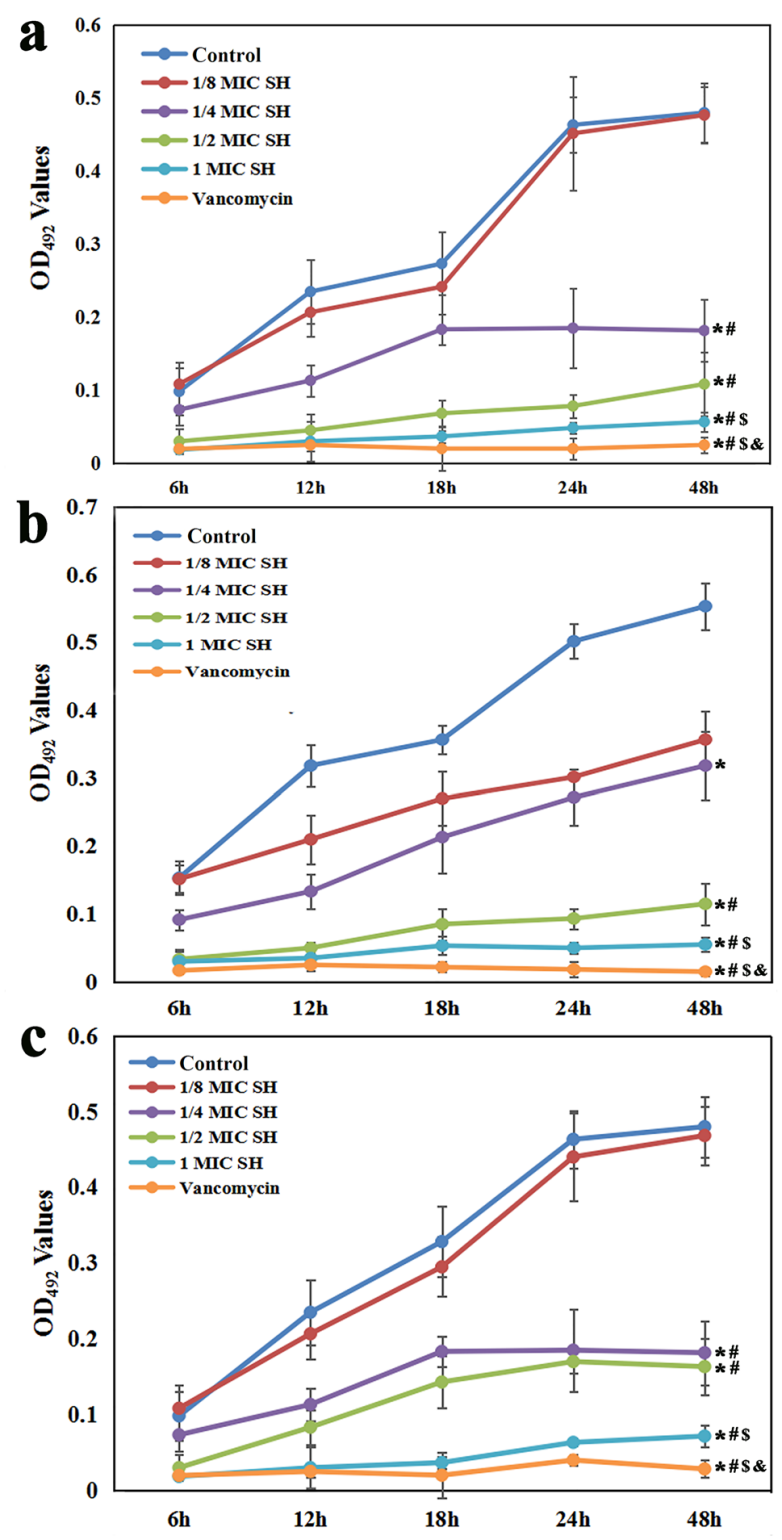
CV staining at different culture time. Compared with the control group, *P < 0.01. Compared with the control and 1/8MIC SH groups, ^#^*P* < 0.01. Compared with 1/4MICl and 1/2MIC SH groups, ^$^*P* < 0.05. Compared with other groups, ^&^*P* < 0.01. (**a**) *S. epidermidis* ATCC 35984. (**b**) *S. aureus* ATCC 43300. (**c**) MRSE 287.

CV staining (Fig. 5) showed that the OD value of biofilm had an increasing trend in the control and 1/8MIC SH groups with the increase of incubation time, suggesting the strains adhered to the titanium surface and gradually formed biofilm; from 6 to 24 h, we observed significant differences of the OD values between any two time-points (*P* < 0.01), while no statistical difference of OD value was found for *S. epidermidis* ATCC 35984 (Fig. 5a) or MRSE 287 (Fig. 5c) (*P* > 0.05) and statistical difference was found for *S. aureus* ATCC 43300 (Fig. 5b) (*P* < 0.01) between 24 and 48 h. For the 1/4MIC SH group of *S. epidermidis* ATCC 35984 (Fig. 5a) and MRSE 287 (Fig. 5c), the OD values increased and difference between any two time-point were found from 6 to 18 h (*P* < 0.01), then no difference from 18 to 48 h (*P* > 0.05); for *S. aureus* ATCC 43300 (Fig. 5b), the OD values of 1/4MIC SH group increased and difference between any two time-point were statistically significant from 6 to 48 h (*P* < 0.05). For the 1/2MIC SH group of *S. epidermidis* ATCC 35984 (Fig. 5a) and *S. aureus* ATCC 43300 (Fig. 5b), the OD values increased and difference between any two time-point were found from 6 to 48 h (*P* < 0.05); for MRSE 287 (Fig. 5c), the OD values of 1/2MIC SH group increased and difference between any two time-point were statistically significant from 6 to 24 h (*P* < 0.01), then no difference from 24 to 48 h (*P* > 0.05). On the contrary, the OD values from 6 to 48 h in the 1MIC SH and vancomycin groups were stable, and no obvious difference was found between any two time-points (*P* > 0.05). At different time point, the OD values of *S. epidermidis* ATCC 35984 (Fig. 5a) and *S. aureus* ATCC 43300 (Fig. 5b) in the control, 1/8MIC SH and 1/4MIC SH groups were higher than that in 1/2MIC SH, 1MIC SH and vancomycin groups (*P* < 0.01). The OD values of MRSE 287 (Fig. 5c) in the control, 1/8MIC SH, 1/4MIC SH and 1/2MIC SH groups were higher than that in 1MIC SH and vancomycin groups (*P* < 0.01). The vancomycin group had the lowest OD value of all the groups (*P* < 0.01).

### SH effects on the expression of *ica *AD and *ica* R

**Figure 6:**
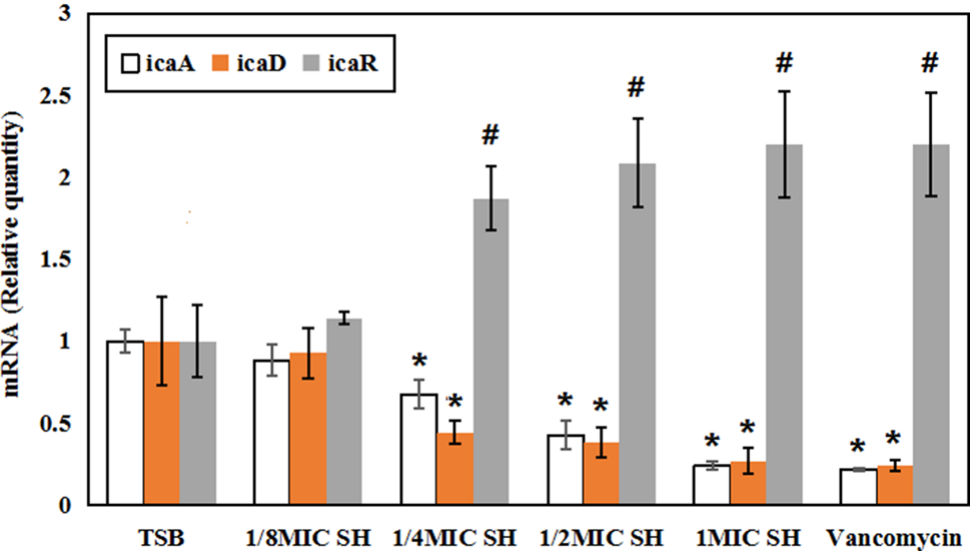
Analysis of ica gene expression (PCR). Compared with TSB and 1/8MIC SH groups, *P < 0.01, ^#^*P* < 0.01.

As shown in Fig. 6, the expression level of *ica* AD was not significantly different between the TSB and 1/8MIC SH groups (*P* > 0.05). The expression level of *ica* AD in the 1/4 MIC, 1/2MIC, 1MIC SH and vancomycin groups decreased compared with that of the TSB and 1/8MIC groups (*P* < 0.05). In the SH groups, the *ica* A expression level showed a gradual decrease with the increase of SH concentration, showing statistically significant differences (*P* < 0.01). Further, no differences of *ica* AD expression were found between the 1MIC SH and vancomycin groups (*P* > 0.05). For *ica* R expression level, there existed a gradual increase with the increase of SH concentration; Compared with the TSB and 1/8MIC SH groups, the 1/4MIC, 1/2MIC, 1MIC SH and vancomycin groups showed high expression level of *ica* R (*P* < 0.01). However, there were no statistically significant differences between the TSB and 1/8MIC SH groups or among the 1/2MIC SH, 1MIC SH and vancomycin groups (*P* > 0.05).

## Discussion

Inhibition of biofilm formation and the killing of antibiotic-resistant bacteria are the key methods for effective treatment of the refractory infections related to the orthopedic implants. Intravenous use of sensitive antibiotics is necessary for the clinical treatment of infections. However, unfortunately, multidrug-resistant bacteria are often found in the bacterial culture of orthopedic infection wounds, and based on the antibiotic sensitivity tests, vancomycin has often become the first choice for clinicians^19^. Studies have shown that only a small portion of intravenous vancomycin can reach bone and joint tissues, and even less penetrates into bone tissues at the site of osteomyelitis; thus, vancomycin may not eliminate multidrug-resistant pathogens in biofilms^20^. In addition, the application of vancomycin has led to the emergence of vancomycin resistant *Staphylococcus* strains^21^.

External application of the TCM, which has been used in the Chinese population to prevent and treat orthopedic infectious for long time, and many prescriptions have been proved to be therapeutically effective^10–12^. According to the literature review, the external application of TCM has beneficial properties such as anti-infection and local immune regulation in the infected wound, which has advantages in the control of acute and chronic orthopedic infections. For local infections of extremities caused by multi-antibiotic-resistant bacteria with exposure of bone and implants, based on the TCM theory of "simmer pus promoting the regeneration", we have successfully controlled the local infected wounds by the external use of SH decoction. Specifically, the infected wound was soaked and washed for 30 min in SH extract twice a day, and after treatment for mean 3 weeks, local infections of the limbs are clearly controlled, secretions and necrotic tissue are reduced, and the granulation tissue grows well, which provided better tissue conditions for reoperation and wound healing. This decoction contains *Scutellaria baicalensis Georgi*, *Coptidis rhizoma*, *Cortex Phellodendri chinsis* and so on, which are heat-clearing and detoxifying drugs. Modern medical research have shown that these Chinese herbs had a broad-spectrum antibacterial and bacteriostatic effects, and no bacterial resistance was found in the combined application. The clinical application of SH prescription can play the role of anti-bacterial and anti-inflammatory in the wound, thus achieving the purpose of eliminating the infections^10–12^.

This study investigated the in vitro effect of SH decoction on adhesion and biofilm formation of bacteria on the titanium surface, and explored the potential value of this prescription as an effective method to treat orthopedic infections. Since the majority of orthopedic implant-related infections are caused by *S. epidermidis* and *S. aureus*^22^. Therefore, we selected the *S. epidermidis* ATCC 35984, *S. aureus* ATCC 43300 and MRSE 287 to understand biofilm formation in this study. Previous research have demonstrated that biofilm formation proceeds in two phases: primary attachment of *Staphylococcal* cells on a biomaterial, which is followed by bacterial accumulation in multiple layers and glycocalyx formation leading to a mature biofilm, the attachment of *Staphylococcus* to titanium surfaces is strongly seen as an essential step in the formation of biofilm^3,7^. If the primary bacterial adhesion is interfered, then the biofilm formation could be prohibited consequently^3,16^. The results in this study showed that SH decoction could inhibit the bacterial adhesion on the titanium surface, and there was a trend that the inhibition effect was more obvious with the increase of dilution concentration of SH decoction. The results also showed that SH decoction could inhibit the formation of dense structural biofilms of the antibiotic-resistant *Staphylococcus* on the titanium surface by either quantitative or qualitative analysis. Specifically, the bacterial strains cannot form biofilms when incubated with the 1MIC of SH decoction, only showing loose adhesion of the bacterial colonies. Although biofilm was formed at 1/4 MIC and 1/2MIC, its OD values were obviously less than that of TSB group. At 1/8MIC of SH decoction, biofilm formation was not inhibited compared to the TSB control group. It can be seen from above analysis that SH decoction could inhibit the biofilm formation on the titanium surface, which is dependent on the dilution concentration of the SH decoction.

Many studies have shown that polysaccharide intercellular adhesin (PIA) is the primary determinant of the accumulation phase of *staphylococcal* biofilm formation^22–24^. It has been reported that the *ica* A and *ica* D partially overlaps in sequence and they together mediate the synthesis of N-acetylglucosamine using UDP-N-acetylglucosamine as a substrate, which composes β-1,6-linked homoglycan to synthesize PIA^25^. Co-expression of the functional *ica* A and *ica* D gene can catalyze the synthesis of PIA, and the down-regulation of *ica* AD expression leads to the reduced activity of N-acetylglucosaminyl transferase, which also leads to reduced amount of PIA^25–27^. In addition, studies have shown that the complex gene regulation system of *Staphylococcus* may ultimately influence the biofilm formation by regulating the expression of *ica* operon^24,27^. Therefore, in order to verify the role of *ica* operon in the inhibition of bacterial biofilm formation on the titanium surface by SH decoction, *S. epidermidis* ATCC 35984 with property of positive biofilm formation and *ica* AD gene expression was selected in this study^13^. The expressions of *ica* AD were detected by real-time fluorescence quantitative PCR, and the results showed that during the process of biofilm formation, the expression level of *ica* A and *ica* D genes in the biofilm and culture medium was inhibited by the SH decoction at different dilution concentrations, and the inhibition became more obvious with the increase of SH concentration. In addition, the expression of *ica* operon is negatively regulated by its upstream inhibitory gene *ica* R^27^. In our study, we found that SH decoction could not only down-regulate the expression of *ica* AD, but also up-regulate the expression level of *ica* R in a concentration dependent manner. Therefore, it could be inferred that the SH decoction activates *ica* R to inhibit the transcription of *ica* AD, thus leading to the reduced synthesis of PIA and formation of biofilm on the titanium surface.

Several limitations of the present study deserve to be mentioned. Firstly, although our results demonstrated a beneficial effect of SH decoction against bacterial biofilm formation, the synergistic effect of SH combined with conventional antibiotics on the bacterial biofifilm remains unclear. Future studies are warranted to clarify this issue. Secondly, the biofilm formed by S*taphylococcus* is a complex process, in which PIA encoded by the *ica* ADBC operon plays a major role^22–24,28^. The complex regulatory system of *Staphylococcus* may ultimately influence the biofilm formation by regulating the expression of *ica* operon. For example, sarA, SigmaB and RsbU genes can promote the transcription level of *ica* ADBC, while *ica* R and lux S can inhibit the transcription of *ica* ADBC^29,30^. Although our study has suggested that SH decoction can inhibit the biofilm formation by down-regulating the expression level of *ica* AD, its effect on the expression of upstream regulatory genes is still unclear. Therefore, additional studies are necessary to better understand these mechanisms.

## Conclusions

SH decoction can inhibit biofilm formation of antibiotic-resistant *Staphylococcus* on the titanium surface based on in vitro results. There appears to be a concentration dependent relationship between SH decoction and biofilm formation. The mechanism may be that the SH decoction inhibit the transcription of *ica* AD, thus, leading to reduced synthesis of PIA and inhibilation of biofilm formation.
